# The contribution of smoking to differences in cardiovascular disease incidence between men and women across six ethnic groups in Amsterdam, the Netherlands: The HELIUS study

**DOI:** 10.1016/j.pmedr.2022.102105

**Published:** 2023-01-02

**Authors:** Renee Bolijn, Mirthe Muilwijk, Mary Nicolaou, Henrike Galenkamp, Karien Stronks, Hanno L. Tan, Anton E. Kunst, Irene G.M. van Valkengoed

**Affiliations:** a Department of Public and Occupational Health, Amsterdam UMC Location University of Amsterdam, Meibergdreef 9, Amsterdam, The Netherlands; b Department of Epidemiology and Data Science, Amsterdam UMC Location Vrije Universiteit Amsterdam, De Boelelaan 1117, Amsterdam, The Netherlands; c Department of Clinical and Experimental Cardiology, Amsterdam UMC Location University of Amsterdam, Meibergdreef 9, Amsterdam, The Netherlands; d Netherlands Heart Institute, Utrecht, The Netherlands

**Keywords:** Cardiovascular disease, Sex and gender differences, Smoking, Ethnicity, HELIUS study

## Abstract

It is unclear to what extent differences in cardiovascular disease (CVD) risk between men and women are explained by differences in smoking, and whether this contribution to risk is consistent across ethnic groups. In this prospective study, we determined the contribution of smoking to differences in CVD incidence between men and women, also in various ethnic groups. We linked baseline data of 18,058 participants of six ethnic groups from the HELIUS study (Amsterdam, the Netherlands) to CVD incidence data, based on hospital admission and death records from Statistics Netherlands (2013–2019). The contribution of smoking to CVD incidence, as estimated by the population attributable fraction, was higher in men than in women, overall (24.1% versus 15.6%) and across most ethnic groups. Among Dutch participants, however, the contribution of smoking was higher among women (21.0%) than men (16.2%). Using Cox regression analyses, we observed that differences in smoking prevalence explained 22.0% of the overall lower hazard for CVD in women compared to men. Smoking contributed minimally to the lower hazards for CVD in women among participants of Dutch (0%), Ghanaian (4.9%) and Moroccan origin (0%), but explained 28.6% and 48.6% of the lower hazards in women in South-Asian Surinamese and African Surinamese groups, respectively. While smoking prevention and cessation may lead to lower CVD incidence in most groups of men and women, it may not substantially reduce disparities in CVD risk between men and women in most ethnic groups.

## Highlights

• Differences in smoking explains 22% of the lower CVD risk in women compared to men.
• In the South-Asian Surinamese, smoking explains 29% of the lower CVD risk in women.
• In the African Surinamese, smoking explains 49% of the lower CVD risk in women.
• In the other ethnic groups, smoking does not explain CVD risk differences.
• Smoking prevention strategies will reduce risk differences in some ethnic groups.

## 1 Introduction

Differences in cardiovascular disease (CVD) risk between men and women have been widely reported across populations (EUGenMed, 2015, Appelman et al., 2015, Mosca et al., 2011), with generally higher incidence rates in men than in women (Albrektsen et al., 2016). The causes for these differences remain not fully resolved, and may include differences in the occurrence of health-related behaviours, such as smoking (Yusuf et al., 2004, Colpani et al., 2018).

Previous studies have reported wide variations in the contribution of smoking to CVD incidence in men and in women separately (e.g. (Anand et al., 2008, Cheng et al., 2014, Nilsson et al., 2006, Schnohr et al., 2002, Trajkova et al., 2017). The extent to which smoking contributes to differences between men and women is less clear. Earlier studies report varying magnitudes of the contribution of smoking to higher CVD incidence in men compared to women, ranging from <10 % to almost 50 % (Albrektsen et al., 2016, Fritz et al., 2015, Huxley et al., 2005, Jousilahti et al., 1999, Tamaki et al., 2006).

Since the prevalence of smoking varies widely across men and women from different ethnic groups within populations (Brathwaite et al., 2017, Salama et al., 2018, Bosdriesz et al., 2013), it is likely that its contribution to differences in CVD incidence between men and women also varies across these groups. Moreover, CVD incidence differs substantially across ethnic groups within populations (Sohail et al., 2015, Agyemang and Van den Born, 2019) and limited evidence suggests that CVD risk differences between men and women may not be consistent across ethnic groups (Ho et al., 2005, Van Oeffelen et al., 2015). Insights into the variations of the contribution of smoking to differences in CVD incidence between men and women across ethnic groups is relevant, as populations are increasingly ethnically diverse, e.g. in the Netherlands (Stoeldraijer et al., 2060). However, previous studies did not assess whether the contribution of smoking to differences in CVD incidence between men and women is consistent across ethnic groups.

Therefore, the aim of this study was to determine the contribution of smoking to differences in CVD incidence between men and women from the population-based, multi-ethnic HEalthy LIfe in an Urban Setting (HELIUS) study, the Netherlands. For this purpose, we first assessed the contribution of smoking to CVD incidence in men and in women separately. We performed this analysis in the overall population and across six ethnic groups. Then, we quantified to what extent differences in CVD incidence between men and women were explained by smoking, both in the overall population and across ethnic groups.

## 2 Methods

We set up a prospective cohort study in which we linked baseline data (i.e., data on smoking status and covariables) from the HELIUS study to data on CVD incidence, based on hospital admission data and death records from Statistics Netherlands.

### 2.1 HELIUS

The HELIUS study is a multi-ethnic prospective cohort study conducted in Amsterdam, the Netherlands, and has been described in detail elsewhere (Snijder et al., 2017, Stronks et al., 2013). Briefly, baseline data collection took place between 2011 and 2015 and included participants of six ethnic groups aged 18–70 years living in Amsterdam. Potential participants were sampled with a simple random sampling method from the municipality registry, after stratification by ethnicity as defined by registered country of birth. Data were obtained by questionnaire and physical examinations were performed (including the collection of biological samples). The HELIUS study has been approved by the AMC Ethical Review Board (MREC 10/100# 17.10.1729). All participants provided written informed consent.

#### 2.1.1 Variables

Sex was derived from the municipality registry and classified as man or woman.

Ethnicity was defined by registered country of birth, combined with the registered parental countries of birth. Participants of Dutch, Surinamese, Ghanaian, Moroccan, and Turkish origin were included. A participant was defined as belonging to one of the included ethnic minority groups if he/she fulfilled one of two criteria: (1) he/she was born outside the Netherlands and has at least one parent born outside the Netherlands (first generation) or (2) he/she was born in the Netherlands but both parents were born outside the Netherlands (second generation). For the Dutch sample, we invited people who were born in the Netherlands and whose parents were born in the Netherlands. After data collection, Surinamese participants were further classified according to self-reported ethnic origin into “African”, “South-Asian”, “Javanese”, or “other”.

Smoking status was based on self-report (‘Do you smoke?’) and classified as current (‘Yes, daily’), former (‘No, but I used to’), and never smoker (‘No, I never smoked’).

Educational level (as domain of socioeconomic status) was based on the self-reported highest qualification attained in the Netherlands or in the country of origin and categorized into four groups: 1) no or elementary, 2) lower vocational or lower secondary, 3) intermediate vocational or intermediate or higher secondary, and 4) higher vocational or university. Family history of CVD was defined by a self-reported CVD diagnosis among first degree family members aged <60 years. Participants were asked to bring their prescribed medications to the research location, which were categorized using the Anatomical Therapeutic Chemical (ATC) classification system. Blood pressure-lowering medication included centrally acting antihypertensives (ATC code C02), diuretics (C03), beta-blockers (C07), calcium channel blockers (C08) and agents acting on the renin–angiotensin–aldosterone system (C09). Glucose-lowering medication was classified as ATC code A10. Lipid-lowering medication was classified as ATC code C10.

### 2.2 Linkage

Using citizen service numbers, baseline data from the HELIUS study were linked to hospital admission data and death records, by Statistics Netherlands. Hospital admission data are provided to Statistics Netherlands by Dutch Hospital Data and include records on all admissions of one day or more to general and academic hospitals in the Netherlands that could be linked to nationwide citizen service numbers (97.3 % in 2013 to 99.7 % in 2019) (Centraal Bureau voor de Statistiek, 2020). Ambulatory contacts (outpatient contacts and emergency room visits without subsequent admission) are not included. For this study, we included all admissions between 2013 and 2019. After linkage, the records were pseudonymized by Statistics Netherlands. We considered admission data as not complete when International Classification of Diseases and Related Health Problems (ICD) codes for primary diagnoses were missing from the record. Completeness of admission data that were linked increased from 93.9 % (2013) to 99.97 % (2016). In2017, 2018, and 2019, records were less complete (75.7 %, 73.1 %, and 74.5 % respectively). This might be related to bankruptcy of a large hospital in Amsterdam in that period.

Death records include all deaths and causes of death of persons registered in the Cause of Death Registry at Statistics Netherlands. Death records were available up to and including 2018.

#### 2.2.1 CVD incidence

CVD incidence was based on 1) first registered hospital admissions for any primary diagnosis of ischaemic heart disease (ICD-10 codes I20-I25), heart failure (I11, I13, I50), cerebrovascular disease (I60-I69), or peripheral artery disease (I70-I79), including aortic aneurysm (I71) after baseline measurement, and on 2) deaths due to any of these diseases, or instantaneous death (R96.0), or death occurring in <24 h from onset of symptoms, not otherwise specified (R96.1) (Jørstad et al., 2016, D’Agostino et al., 2008). For sensitivity analyses, we additionally included hypertensive disease (I10-I15) and cardiac arrhythmias (I44-I49) (Conroy et al., 2003).

### 2.3 Study population

Of the 22,165 participants with available questionnaire data and data from physical examinations, 19,932 participants gave permission for data linkage and were included in this study (Supplemental Fig. S1). Linkage was successful for 19,893 participants (99.8 %). We excluded participants of Javanese Surinamese (n = 218) or unknown Surinamese (n = 237) origin and those with another/unknown ethnic origin (n = 44), due to low statistical power. Then, we excluded participants with a history of CVD at baseline (n = 1,039) or missing data on prior CVD (n = 297), based on self-reported prior myocardial infarction, cerebrovascular accident, angioplasty or bypass surgery on heart or legs. This resulted in a study population of 18,058 participants: 7,645 men and 10,413 women.

### 2.4 Statistical analyses

Baseline characteristics were stratified by sex, and presented as arithmetic means and standard deviations (numerical data) or percentages and frequencies (categorical data). Distributions of smoking status were also stratified by ethnicity.

Crude and age-standardized CVD incidence rates per 1,000 person-years were calculated for men and women, overall and by ethnicity. Follow-up duration was determined from inclusion date within HELIUS until first CVD event, death, or end of follow-up duration (31 December 2019). Participants were lost to follow-up in the case of emigration, but we do not know how many participants this pertains. For age-standardization, we used the standard age structure of the World Health Organization world population (Ahmad et al., 2001).

First, we examined the contribution of smoking to CVD incidence in men and in women overall (main analyses) and across ethnic groups. We conducted Cox proportional hazards (PH) regression analyses to determine the hazard ratio (HR) of the smoking categories for CVD incidence in the overall population. These analyses were adjusted for sex, age, ethnicity, educational level, and family history of CVD. An interaction term for sex and smoking was checked but not included as it did not significantly improve the model (as determined by likelihood ratio test). The PH assumption was checked by visual inspection and tests based on Schoenfeld residuals. Violations for covariates were solved by stratification for the variable (categorical variables) or by adding an interaction term with time (continuous variable). Since there were only few missings in study population characteristics (<5%; Table 1, Supplemental Table S1), we performed complete case analysis. Then, we estimated the population attributable fraction (PAF) for smoking status in men and in women using the following formula for multicategory exposures: PAF = (1 − ∑ (P/HR)) × 100 (Rockhill et al., 1998). P is the prevalence of the smoking categories among incident CVD cases in men and in women overall, and stratified by ethnicity.

**Table 1:** Distribution of smoking status and crude and age-standardized CVD incidence rates, stratified by sex and ethnicity.

	Men (n, %)	Women (n, %)
*Overall*	*n = 7,645*	*n = 10,413*
Smoking status		
Current	2,388 (31.2)	1,853 (17.8)
Former	1,961 (25.7)	1,656 (15.9)
Never	3,271 (42.8)	6,874 (66.0)
Missing	25 (0.3)	30 (0.3)
CVD cases (n)	227	190
Crude CVD incidence, per 1,000 person-years	5.1	3.1
Age-standardized CVD incidence, per 1,000 person-years	3.9 (3.4–4.4)	2.5 (2.1–2.9)

*Dutch*	*n = 1,878*	*n = 2,225*
Smoking status		
Current	485 (25.8)	508 (22.8)
Former	729 (38.8)	814 (36.6)
Never	662 (35.3)	896 (40.3)
Missing	2 (0.1)	7 (0.3)
CVD cases (n)	60	28
Crude CVD incidence, per 1,000 person-years	5.6	2.2
Age-standardized CVD incidence, per 1,000 person-years	3.9 (2.8–4.9)	1.4 (0.9–1.9)

*South-Asian Surinamese*	*n = 1,073*	*n = 1,415*
Smoking status		
Current	408 (38.0)	255 (18.0)
Former	174 (16.2)	154 (10.9)
Never	487 (45.4)	1,003 (70.9)
Missing	4 (0.4)	3 (0.2)
CVD cases (n)	55	52
Crude CVD incidence, per 1,000 person-years	8.6	6.1
Age-standardized CVD incidence, per 1,000 person-years	7.1 (5.1–9.0)	4.0 (2.9–5.2)

*African Surinamese*	*n = 1,362*	*n = 2,092*
Smoking status		
Current	572 (42.0)	496 (23.7)
Former	288 (21.1)	385 (18.4)
Never	494 (36.3)	1,205 (57.6)
Missing	8 (0.6)	6 (0.3)
CVD cases (n)	47	46
Crude CVD incidence, per 1,000 person-years	5.9	3.7
Age-standardized CVD incidence, per 1,000 person-years	3.4 (2.4–4.5)	2.8 (1.8–3.8)

*Ghanaian*	*n = 763*	*n = 1,208*
Smoking status		
Current	54 (7.1)	28 (2.3)
Former	99 (13.0)	57 (4.7)
Never	609 (79.8)	1,119 (92.6)
Missing	1 (0.1)	4 (0.3)
CVD cases (n)	18	12
Crude CVD incidence, per 1,000 person-years	3.7	1.6
Age-standardized CVD incidence, per 1,000 person-years	2.5 (1.1–3.9)	2.1 (0.2–4.1)

*Turkish*	*n = 1,311*	*n = 1,569*
Smoking status		
Current	533 (40.7)	463 (29.5)
Former	330 (25.2)	184 (11.7)
Never	441 (33.6)	916 (58.4)
Missing	7 (0.5)	6 (0.4)
CVD cases (n)	27	32
Crude CVD incidence, per 1,000 person-years	3.6	3.6
Age-standardized CVD incidence, per 1,000 person-years	3.9 (1.9–5.9)	4.5 (2.6–6.3)

*Moroccan*	*n = 1,258*	*n = 1,904*
Smoking status		
Current	336 (26.7)	103 (5.4)
Former	341 (27.1)	62 (3.3)
Never	578 (45.9)	1,735 (91.1)
Missing	3 (0.2)	4 (0.2)
CVD cases (n)	20	20
Crude CVD incidence, per 1,000 person-years	2.9	1.9
Age-standardized CVD incidence, per 1,000 person-years	2.7 (1.5–3.8)	1.9 (1.0–2.9)

CVD, cardiovascular disease.

Second, we quantified the contribution of smoking to differences in CVD incidence between men and women, overall (main analyses) and across ethnic groups, also using Cox PH regression analyses. In the first model (model 1), we examined the association between sex and CVD incidence, adjusted for age, ethnicity (not in stratified analyses), educational level, and family history of CVD. Then, we added smoking (model 2) and calculated the relative change of the new HR (HR_2_) for sex after adjustment for smoking compared to the HR from the first model (HR_1_) by using the following formula: (HR_1_ – HR_2_)/(HR_1v_− 1) × 100 %.

Since differences in CVD risk between men and women may differ by age, we explored whether the contribution of smoking to CVD incidence in men and in women and to differences between men and women was consistent across age-groups (<50 and ≥50 years). The cut-off at 50 years was chosen because of the increased CVD risk for women after menopause transition (El Khoudary et al., 2020).

We performed several sensitivity analyses. For PAF analyses, we explored a model with additional adjustments for use of antihypertensive medication, glucose-lowering medication, and lipid-lowering medication, but since estimates hardly altered, we do not show the data. Furthermore, since the awareness of certain risk may influence health-related behaviours and its associations with CVD incidence, we repeated the main PAF analyses restricted to participants without self-reported hypertension or diabetes. In addition, we repeated all main analyses with the broader CVD definition (including hypertensive disease and cardiac arrhythmias).

All statistical analyses were performed in R studio version 3.6.2 (RStudio Team, 2016). P-values <0.05 were regarded as statistically significant.

## 3 Results

Mean age at baseline was 44.4 years in men and 43.6 years in women (Supplemental Table S1). Men were more often current smokers (31.2 %) than women (17.8 %; Table 1). The distribution of smoking in women versus men varied substantially across ethnic groups. For instance, while the prevalence of current smoking was consistently higher among men compared to women in ethnic minority groups, the prevalence was more similar among men and women of Dutch origin.

**Fig. 1:**
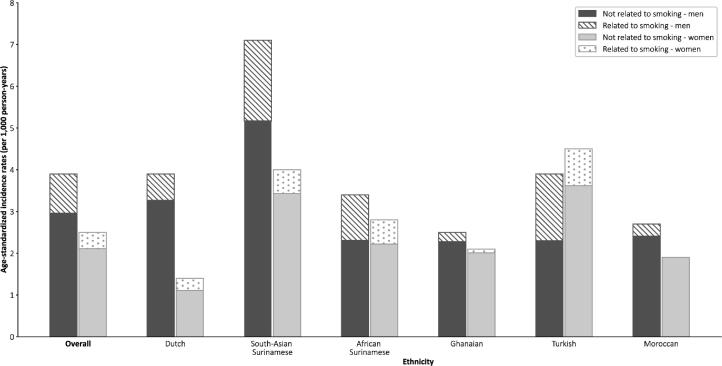
Age-standardized CVD incidence rates split for the proportions related to smoking and not related to smoking, by sex, overall and per ethnic group.

After a mean follow-up duration of 5.8 years (standard deviation 1.3 years) in both men and women, 227 men and 190 women had a CVD event, corresponding to age-standardized CVD incidence rates of 3.9 and 2.5 per 1,000 person-years, respectively (Table 1). One third of men and 16 % of women had an acute myocardial infarction as first event, whereas around 20 % of both men and women had a stroke as first event (Supplemental Table S2). CVD incidence rates were higher among men than women across most ethnic groups, except in the Turkish group (Fig. 1, Table 1).

Current smoking was significantly associated with a higher hazard for CVD, compared to never smoking (HR 2.10, 95 % confidence interval [CI] 1.65–2.67; Table 2). The contribution of smoking ranged from 8.8 % among Ghanaian men to 40.9 % among Turkish men and from 0.1 % among Moroccan women to 21.0 % among Dutch women. The contribution of smoking to CVD incidence was higher in men than in women, overall (24.1 % versus 15.6 %) and across most ethnic groups (Fig. 1, Table 2). Among the Dutch participants, smoking contributed more in women (21.0 %) than in men (16.2 %). The difference in incidence rates without the contribution of smoking between men and women appeared smaller compared to the difference in rates with the contribution of smoking (Fig. 1). This was observed in most ethnic groups (e.g., African Surinamese), but not in the Turkish.

**Table 2:** Association of smoking status with CVD incidence (hazard ratios) and its contribution to CVD incidence (PAF) in men and in women, stratified by ethnicity.

		Men (n = 7,645)	Women (n = 10,413)
	HR (95 % CI)^a^	Distribution of incident CVD cases (%)	Distribution of incident CVD cases (%)
*Smoking status*			
Current	**2.10 (1.65–2.67)**	45.3	29.5
Former	1.01 (0.77–1.34)	26.2	14.2
Never	1.00 (reference)	28.4	56.3

		**PAF**^b^	**PAF**^b^
Overall		24.1	15.6
Dutch		16.2	21.0
South-Asian Surinamese		27.2	14.2
African Surinamese		32.1	20.7
Ghanaian		8.8	4.3
Turkish		40.9	19.6
Moroccan		10.8	0.1

CVD, cardiovascular disease; CI, confidence interval; HR, hazard ratio; PAF, population attributable fraction.

Statistically significant associations (p < 0.05) are printed in bold.

a Adjusted for sex, age, ethnicity, educational status, and family history of CVD.

b PAF = (1 − ∑ (P/HR)) × 100.

**Fig. 2:**
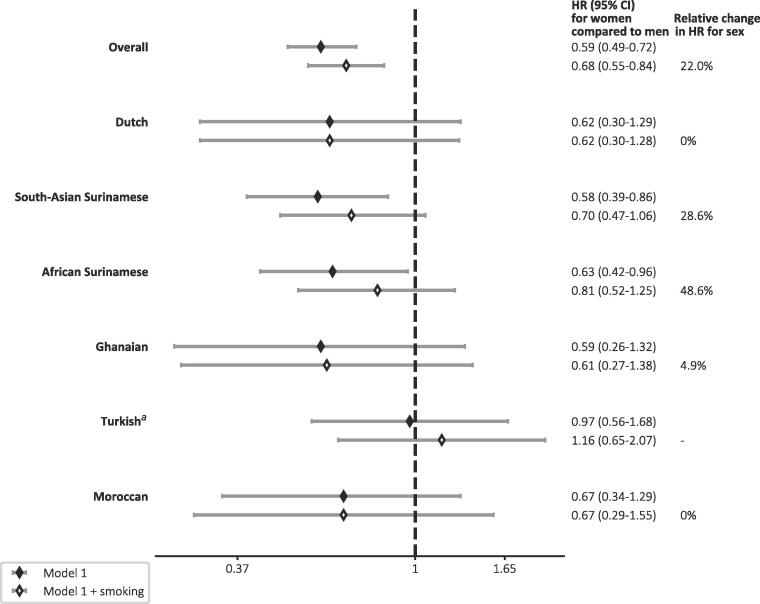
Forest plot of adjusted hazard ratios (HRs) for CVD incidence of women compared to men and relative change in HR for sex after additional adjustment for smoking, overall and per ethnic group. ^a^Proportional hazards assumption violated. Estimates should thus be treated with caution.

After adjustments for age, ethnicity, educational level, and family history of CVD, women had an overall lower hazard for CVD than men (HR 0.59, 95 % CI 0.49–0.72; Fig. 2). Differences in smoking explained 22.0 % of the lower hazard in women (smoking adjusted HR 0.68, 95 % CI 0.55–0.84). Differences in the hazard for CVD between men and women were in similar direction across ethnic groups (range HR: 0.58–0.67 for all groups except Turkish in whom PH assumption was not met), but the contribution of smoking to these differences varied substantially across ethnic groups. While smoking did not or scarcely change the HR for sex on top of adjustments for age, educational level, and family history of CVD among participants of Dutch (0 %), Ghanaian (4.9 %) and Moroccan origin (0 %), 28.6 % and 48.6 % of the lower hazard in women was explained by smoking in South-Asian Surinamese and African Surinamese groups, respectively.

Across age-groups, smoking also contributed consistently more to CVD incidence in men than women (Supplemental Table S3). Smoking contributed slightly more to differences between men and women among participants aged <50 years than among those aged ≥50 years.

Our findings were not substantially different in the sensitivity analyses restricted to participants without self-reported hypertension or diabetes (Supplemental Table S4), or with the broader definition of CVD (Supplemental Tables S5 and S6).

## 4 Discussion

Overall and across most ethnic groups, smoking contributed more to CVD incidence in men than in women, except among the Dutch. However, smoking explained only a part of the differences in CVD risk between men and women in the overall population. Moreover, it did so only in some ethnic groups, particularly in the South-Asian Surinamese and African Surinamese groups.

The large multi-ethnic sample size of our study enabled us to stratify our analyses for both sex and ethnicity. However, our study also has limitations. First, due to the young age of the study population (average age at baseline ≈45 years) and a short follow-up duration (≈6 years), the CVD event rate and the total number of events were low. Thus, our stratified analyses possibly had limited power to demonstrate associations. Moreover, generalizability of our findings to older populations may be limited, since 1) women tend to develop CVD at an older age, and risk differences between men and women decrease with increasing age (Albrektsen et al., 2016, George et al., 2015), and 2) changes in relative differences in prevalence rates of smoking between men and women across decades possibly influence the contribution of smoking to risk differences between men and women. For instance, in the Netherlands, smoking prevalence was higher among men (35 %) compared to women (26 %) in 1980, but declined to more similar rates between men (22 %) and women (20 %) in 2012 (Ng et al., 2014). Second, we may have missed CVD cases in our study population, as hospital admission data were not complete. However, the percentage of missings in hospital admission data did not differ substantially between men and women (13.0 % and 13.1 %) and between ethnic groups (range: 11.1–16.5 %, and low percentage among the Ghanaians: 6.5 %), making it unlikely that our results were influenced. Third, our analyses may have suffered from differential lost to follow-up due to emigration, potentially underestimating CVD incidence more so in ethnic minority groups than in the Dutch majority group. If men and women in these groups did not emigrate equally, this may have influenced our findings. Finally, our measure of smoking may be suboptimal. For instance, self-reported behaviours may introduce social desirability bias. In addition, smoking status was only measured once, potentially resulting in underestimation of the associations (regression dilution bias) (MacMahon et al., 1990).

Overall, more than 20 % of the lower risk of CVD in women compared to men was explained by differences in smoking, consistently across age-groups. Our findings are in line with some studies (Huxley et al., 2005, Jousilahti et al., 1999), but not with others which reported that contributions of smoking to differences between men and women were <10 % (Albrektsen et al., 2016, Fritz et al., 2015) or almost 50 % (Tamaki et al., 2006). This is largely due to substantial differences in smoking prevalence between men and women across studies, although direct comparison between studies is challenging due to differences in, for instance, CVD outcomes and statistical analyses.

It has been suggested that women who smoke may be at higher risk for CVD than men who smoke, potentially due to the greater absorption of toxic agents from cigarettes (Huxley and Woodward, 2011). However, similar to previous studies on the contribution of smoking to differences in CVD risk between men and women (Huxley et al., 2005, Jousilahti et al., 1999, Tamaki et al., 2006), we did not observe a differential impact of smoking on CVD risk for men and women in our study population, as supported by a non-significant interaction term for sex and smoking. Thus, the overall excess CVD risk among men appears to be the result of the higher prevalence of smoking among men, leading to the higher contribution of smoking to CVD incidence in men compared to women.

When we stratified our findings by ethnicity, the contribution of smoking to differences between men and women was largely driven by the South-Asian Surinamese and African Surinamese groups. Within these groups, around 29 % and 49 % of differences between men and women was explained by differences in smoking. In the Turkish group, women had a higher CVD incidence than men and this excess risk for Turkish women appears to be larger without the contribution of smoking, as is also suggested by the larger HR after adjustment for smoking (although the HRs should be cautiously interpreted due to violation of the PH assumption). There may be several reasons why smoking is only relevant for differences between men and women in some of the ethnic groups, particularly the South-Asian Surinamese and African Surinamese groups. First, the overall prevalence of smoking is relatively high in these groups compared to, for instance, the Ghanaian and Moroccan groups. These differences in smoking rates across ethnic groups within populations may reflect differences in cultural and socioeconomic factors related to both pre- and post-migration environments (Brathwaite et al., 2017, Salama et al., 2018, Bosdriesz et al., 2013). Second, relative differences in smoking prevalence between men and women seem larger in most ethnic minority groups (e.g. 38 % versus 18 % in South-Asian Surinamese) compared to the Dutch majority population (26 % versus 23 %). This is in line with previous studies from Finland and the USA reporting on larger differences in prevalence rates of smoking between men and women from ethnic minority groups compared to the more similar rates among men and women from majority populations (Salama et al., 2018, Bosdriesz et al., 2013). As suggested by a previous study based on the HELIUS population, acculturation may already have resulted in convergence of smoking rates among ethnic minority groups towards smoking rates of the Dutch majority population, particularly in women, leading to smaller differences between men and women compared to the countries of origin (Brathwaite et al., 2017). Further acculturation may lead to more similar smoking rates between men and women among ethnic minority groups.

Comparisons between European studies indicate that changes towards more similar prevalence rates of smoking among men and women of majority populations over time have influenced the contribution of smoking to CVD risk differences between men and women. For instance, our findings of the lack of contribution of smoking to risk differences between men and women in the Dutch majority population are similar to the findings of two recent studies in ethnically homogenous populations in European high income countries, reporting a small contribution of smoking to risk differences (Albrektsen et al., 2016, Fritz et al., 2015). On the contrary, a larger contribution of smoking was reported in an older study conducted in Europe, which also reported larger differences in prevalence rates between men and women (Jousilahti et al., 1999).

In conclusion, our findings indicate that some of the differences in CVD risk between men and women, overall and particularly in South-Asian Surinamese and African Surinamese participants, were explained by differences in smoking. Considering the substantial contribution of smoking to CVD incidence in men and women of most ethnic groups, we recommend to further encourage smoking prevention and cessation across these groups, potentially through more ethnic- and gender-specific programs (Smith et al., 2016, Thomas et al., 2015, Webb Hooper et al., 2015). However, this strategy will only reduce disparities in CVD risk between men and women in South-Asian Surinamese and African Surinamese groups. This indicates that future research on disparities in CVD risk between men and women may focus on other health-related behaviours, such as alcohol consumption, as well as on factors related to access to and use of (preventive) treatment, or to exposure to psychosocial stressors (Mauvais-Jarvis et al., 2020, O’Neil et al., 2018, Connelly et al., 2021).

## CRediT authorship contribution statement

**Renee Bolijn:** Conceptualization, Methodology, Formal analysis, Data curation, Writing – original draft, Visualization. **Mirthe Muilwijk:** Conceptualization, Methodology, Writing – review & editing. **Mary Nicolaou:** Writing – review & editing. **Henrike Galenkamp:** Validation, Writing – review & editing. **Karien Stronks:** Writing – review & editing. **Hanno L. Tan:** Methodology, Writing – review & editing. **Anton E. Kunst:** Methodology, Writing – review & editing. **Irene G.M. van Valkengoed:** Conceptualization, Methodology, Writing – review & editing, Supervision, Project administration, Funding acquisition.

## Declaration of Competing Interest

The authors declare that they have no known competing financial interests or personal relationships that could have appeared to influence the work reported in this paper.

## Data Availability

Data will be made available on request.
