# Supplementary figures and images for: The contribution of smoking to differences in cardiovascular disease incidence between men and women across six ethnic groups in Amsterdam, the Netherlands: The HELIUS study
Source: Prev Med Rep. 2023 Jan 2;31:102105. doi: 10.1016/j.pmedr.2022.102105 (PMC9938300; doi:10.1016/j.pmedr.2022.102105)

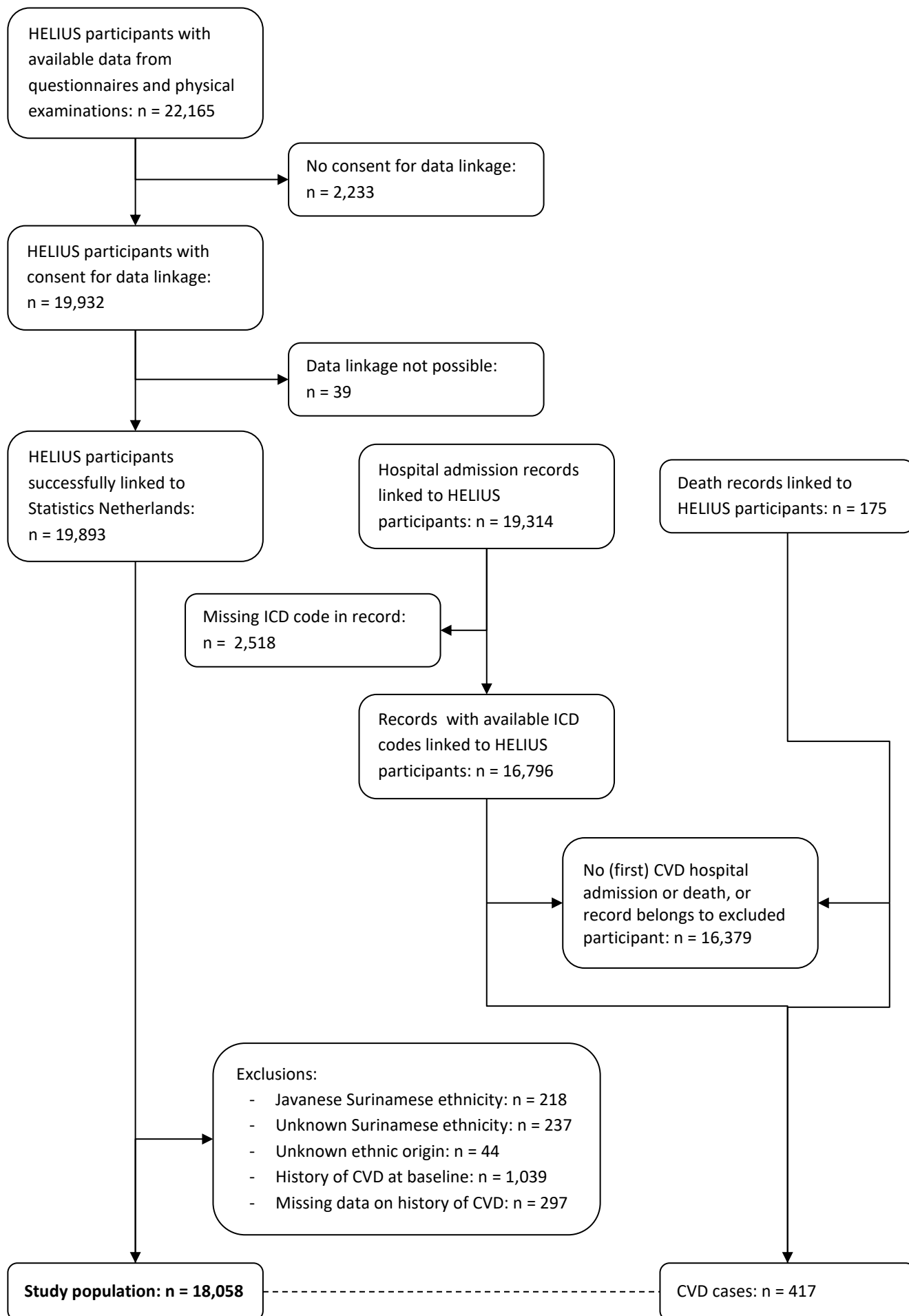

**Supplemental Figure 1.** Flow diagram of the study population

Supplement: Supplementary data 1 [file mmc1.pdf]
