# Supplementary material for: The contribution of smoking to differences in cardiovascular disease incidence between men and women across six ethnic groups in Amsterdam, the Netherlands: The HELIUS study
Source: Prev Med Rep. 2023 Jan 2;31:102105. doi: 10.1016/j.pmedr.2022.102105 (PMC9938300; doi:10.1016/j.pmedr.2022.102105)
Supplement: Supplementary data 2 [file mmc2.pdf]

Supplemental Table 1. Characteristics of the study population, stratified by sex

	**Men (n=7,645)**	**Women (n=10,413)**
Age at baseline, in years	44.4 (13.2)	43.6 (13.2)
Age-groups		
<50 years	4,590 (60.0)	6,522 (62.6)
≥50 years	3,055 (40.0)	3,891 (37.4)
Ethnicity		
Dutch	1,878 (24.6)	2,225 (21.4)
South-Asian Surinamese	1,073 (14.0)	1,415 (13.6)
African Surinamese	1,362 (17.8)	2,092 (20.1)
Ghanaian	763 (10.0)	1,208 (11.6)
Turkish	1,311 (17.1)	1,569 (15.1)
Moroccan	1,258 (16.5)	1,904 (18.3)
Educational level		
Never or elementary only	1,016 (13.3)	2,051 (19.7)
Low	2,145 (28.1)	2,524 (24.2)
Intermediate	2,282 (29.8)	3,011 (28.9)
High	2,151 (28.1)	2,749 (26.4)
Missing	51 (0.7)	78 (0.7)
Family history of CVD		
Yes	1,563 (20.4)	2,417 (23.2)
No	5,073 (66.4)	6,856 (65.8)
Do not know	951 (12.4)	1,018 (9.8)
Missing	58 (0.8)	122 (1.2)
Self-reported hypertension	2,891 (37.8)	3,319 (31.9)
Missing	15 (0.2)	30 (0.3)
Use of blood pressure-lowering medication	936 (12.2)	1,731 (16.6)
Self-reported diabetes	618 (8.1)	809 (7.8)
Missing	25 (0.3)	69 (0.7)
Use of glucose-lowering medication	478 (6.3)	665 (6.4)
Use of lipid-lowering medication	664 (8.7)	752 (7.2)

Data are presented as means (standard deviations) or frequencies (percentages).

CVD, cardiovascular disease.

Supplemental Table S2. Frequency of specific cardiovascular diseases (including ICD-10 codes), by sex

	**Men (n, %)**	**Women (n, %)**
Angina pectoris (I20)	45 (19.8)	67 (35.3)
Acute myocardial infarction (I21)	66 (29.1)	30 (15.8)
Stroke (I60-I64)	51 (22.5)	38 (20.0)
Other, including heart failure and peripheral artery disease	65 (28.6)	55 (28.9)
**Total**	**227**	**190**

ICD, International Classification of Diseases.

Supplemental Table S3. Contribution of smoking to CVD incidence in men and in women (PAF), and hazard ratios for CVD incidence of women compared to men and relative change in hazard ratio for sex after additional adjustment for smoking, stratified by age-groups

	PAF[a]		Women versus men			
	**Men**	**Women**		**HR (95% CI)**	**p-value**	**Relative change in HR for sex[c]**
Age <50 years (n=11,112)	30.5	13.2	Model 1[b]	0.67 (0.45-1.01)	0.06	
			Model 1 + smoking	0.76 (0.50-1.15)	0.20	27.3%
Age ≥50 years (n=6,946)	22.1	16.4	Model 1[b]	**0.57 (0.45-0.72)**	**<0.001**	
			Model 1 + smoking	**0.66 (0.52-0.84)**	**<0.001**	20.9%

CVD, cardiovascular disease; CI, confidence interval; HR, hazard ratio; PAF, population attributable fraction.

[a] PAF = (1 - ∑ (P / HR)) x 100

[b] Adjustments: age, ethnicity, educational status, family history of CVD.

[c] $(HR^1 – HR^2) / (HR^1 – 1) \times 100\%$

Supplemental Table S4. Association of smoking status with CVD incidence (hazard ratios), and its contribution to CVD incidence (PAF) in men and women without self-reported hypertension or diabetes

	Men (n=4,536)			Women (n=6,768)	
	**HR (95% CI)[a]**	**Distribution of incident CVD cases (%)**	**PAF[b]**	**Distribution of incident CVD cases (%)**	**PAF[b]**
Smoking status					
Current	**1.96 (1.25-3.07)**	46.0		38.5	
Former	0.97 (0.56-1.67)	25.4	21.7	x[c]	18.3
Never	1.00 (reference)	28.6		x[c]	

CVD, cardiovascular disease; CI, confidence interval; HR, hazard ratio; PAF, population attributable fraction.

Statistically significant associations ($p<0.05$) are printed in bold.

[a] Adjusted for sex, age, ethnicity, educational status, and family history of CVD.

[b] $PAF = 1 - \sum (P / HR) \times 100$

[c] Prevalence not reported due to small numbers (<10 cases) in former smokers group. Prevalence of never smoking also not reported to avoid calculation of the prevalence of former smokers.

Supplemental Table S5. Association (hazard ratios) of smoking status with CVD incidence (broader definition, including hypertensive disease and cardiac arrhythmias), and its contribution to CVD incidence (PAF) in men and in women

	Men (n=7,645)			Women (n=10,413)	
	**HR (95% CI)[a]**	**Distribution of incident CVD cases (%)**	**PAF[b]**	**Distribution of incident CVD cases (%)**	**PAF[b]**
Smoking status					
Current	**1.75 (1.41-2.18)**	39.8		25.0	
Former	1.16 (0.92-1.48)	32.6	21.6	16.3	13.0
Never	1.00 (reference)	27.6		58.7	

CVD, cardiovascular disease; CI, confidence interval; HR, hazard ratio; PAF, population attributable fraction.

Statistically significant associations ($p<0.05$) are printed in bold.

[a] Adjusted for sex, age, ethnicity, educational status, and family history of CVD.

[b] $PAF = (1 - \sum (P / HR)) \times 100$

Supplemental Table S6. Hazard ratios for CVD incidence (broader definition, including hypertensive disease and cardiac arrhythmias) of women compared to men, and relative change in hazard ratio for sex after additional adjustment for smoking (n=18,058)

	**Women versus men**		
	**HR (95% CI)**	**p-value**	**Relative change in HR for sex[b]**
Model 1[a]	0.64 (0.54-0.77)	<0.001	
Model 1 + smoking	0.72 (0.60-0.87)	<0.001	22.2%

CVD, cardiovascular disease; CI, confidence interval; HR, hazard ratio.

[a] Adjustments: age, ethnicity, educational status, and family history of CVD.

[b] $(HR^1 – HR^2) / (HR^1 – 1) \times 100\%$
